# Supercritical CO_2_ Processing of a Functional Beverage Containing Apple Juice and Aqueous Extract of *Pfaffia glomerata* Roots: Fructooligosaccharides Chemical Stability after Non-Thermal and Thermal Treatments

**DOI:** 10.3390/molecules25173911

**Published:** 2020-08-27

**Authors:** Eric Keven Silva, Matheus A. Bargas, Henrique S. Arruda, Renata Vardanega, Glaucia M. Pastore, M. Angela A. Meireles

**Affiliations:** 1 LASEFI, Department of Food Engineering, School of Food Engineering, University of Campinas, Campinas 13083-862, Brazil; engerickeven@gmail.com (E.K.S.); mathbargas@gmail.com (M.A.B.); renatavardanega@gmail.com (R.V.); 2 Bioflavors and Bioactive Compounds Laboratory, Department of Food Science, School of Food Engineering, University of Campinas, Campinas 13083-862, Brazil; hsilvanoarruda@gmail.com (H.S.A.); glaupast@unicamp.br (G.M.P.)

**Keywords:** dietary fibers, prebiotic carbohydrate, 1-kestose, fructose, beta-ecdysone, pasteurization, sugar hydrolysis, emerging technology, green chemistry

## Abstract

The effects of supercritical CO_2_ processing on the chemical stability of fructooligosaccharides (FOS) and other functional and nutritional compounds were evaluated employing non-thermal and thermal approaches. Apple juice was enriched with *Pfaffia glomerata* roots aqueous extract due to its high content of short-chain FOS and then subjected to different levels of temperature (40 and 60 °C), pressure (8 and 21 MPa), and CO_2_ volume ratio (20 and 50%). The percentage of CO_2_ volume was evaluated concerning the total volume of the high-pressure reactor. Also, the functional beverage was thermally treated at 105 °C for 10 min. Physicochemical properties (pH and soluble solid content), beta-ecdysone, sugars (glucose, fructose, and sucrose), and FOS (1-kestose, nystose, and fructofuranosylnystose) content were determined. The pH and soluble solid content did not modify after all treatments. The pressure and CO_2_ volume ratio did not influence the FOS content and their chemical profile, however, the temperature increase from 40 to 60 °C increased the nystose and fructofuranosylnystose content. High-temperature thermal processing favored the hydrolysis of 1-kestose and reduced the sucrose content. Regarding beta-ecdysone, its content remained constant after all stabilization treatments demonstrating thus its high chemical stability. Our results demonstrated that supercritical CO_2_ technology is a promising technique for the stabilization of FOS-rich beverages since the molecular structures of these fructans were preserved, thus maintaining their prebiotic functionality.

## 1. Introduction

The supplementation of food products and beverages with prebiotic carbohydrates, also known as fermentable dietary fibers has increased worldwide in the last years due to a unique lifestyle based on the intake of non-digestible compounds able to stimulate the growth/activity of beneficial bacteria in the human gastrointestinal tract [1]. Health disorders like high cholesterol, high blood sugar, weight gain, allergies, and others may be associated with an imbalance in the gut microbiome [2,3]. In this sense, prebiotic carbohydrates, such as fructooligosaccharides (FOS) and inulin type-fructans, may assist in regaining and maintaining health because these macromolecules feed probiotic bacteria, known as *Bifidobacteria*, which are essential nutrients and a source of energy [4]. The gut microbiota modulation by increasing the *Bifidobacteria* count favors beneficial health effects such as the inhibition of pathogens and harmful bacteria that colonize and/or infect the gut mucosa, regulation of intestinal microbial homeostasis, bioconversion of dietary compounds in short-chain fatty acids, production of vitamins, improvements of local and systemic immune responses, and others [5,6].

FOS are well-known fructan oligosaccharides with a linear structure of fructose units linked by β-(2→1) bonds presenting a d-glucose residue at the terminal, reducing end by an α-(1→2) bond. Generally, these oligosaccharides present a degree of polymerization, varying from three to nine fructose molecules [7]. They are widely distributed in a variety of plants as a storage carbohydrate [8]. Several products have been enriched with these prebiotic compounds to increase their functionality like semi-hard cheese [9], fermented sausages [10], Greek yogurt [11], fruit juices [12], infant formulas [13], and many others. Recently Brazilian ginseng (*Pfaffia glomerata*) roots were recognized as a new source of short-chain FOS, including 1-kestose, nystose, and fructofuranosylnystose with up to 8.8 g/100 g of prebiotic carbohydrates [14].

Furthermore, *Pfaffia glomerata* roots are rich in beta-ecdysone, containing up to 0.7/100 g. This ecdysteroid is used in folk medicine due to its analgesic and anti-inflammatory activity. Furthermore, it is recommended to treat memory diseases as well as a tonic [15]. In this sense, the formulation of a novel beverage based on the addition of *Pfaffia glomerata* root extracts to a fruit or vegetable juices as a carrier liquid medium would be a promising health-promoting food product. However, the use of thermal treatments in pasteurization conditions of fruit juices and beverages has promoted the FOS hydrolysis/depolymerization [16,17]. Other functional compounds like ascorbic acid and anthocyanins also are degraded in thermally treated food products. Conventional thermal treatments applied to food products may reduce their sensory qualities due to the formation of off-flavors and “cooked taste” in plant-based beverages. Thus, one challenge for the food industries in the next years is to ensure the safety of food products preserving their functional compounds and sensory attributes similar to unprocessed products. [18].

Innovative technologies to stabilize food and beverages have emerged as potential alternatives to conventional thermal processing applied to inactivate the pathogenic and spoilage microorganisms besides endogenous enzymes associated with the degradation of food products during their storage and shelf life. Many of these have a non-thermal process approaches such as pulsed electric field [19], high-intensity ultrasound [20], high-pressure processing [21], ultraviolet-C light [22], and supercritical carbon dioxide (SC-CO_2_) technology [23]. The chemical stability of prebiotic carbohydrates like FOS and other bioactive compounds like beta-ecdysone after stabilization treatments based on emerging technology is an important research issue due to different physical and chemical mechanisms associated with these. Also, after stabilization treatments, chemical structures must be monitored to ensure that all functionalities expected from plant material are retained. Among innovative technologies, the use of SC-CO_2_ was highlighted as a promising technique for the non-thermal processing of fruit and vegetable juices. In a non-thermal way applying a pressure range of 8 to 60 MPa, SC-CO_2_-stabilized plant-based juices presented sensorial characteristics similar to the fresh-like product maintaining their nutritional value and physicochemical characteristics very close to the untreated juices [24]. Thus, SC-CO_2_ technology could be an interesting alternative to stabilize a new functional beverage using the valuable compounds obtained from *Pfaffia glomerata* roots. Therefore, this study aimed to evaluate the effect of SC-CO_2_ technology on the chemical stability of FOS after non-thermal (40 °C) and thermal (60 °C) stabilization treatments of a functional beverage containing apple juice and aqueous extract of *Pfaffia glomerata* roots. SC-CO_2_ treatments were compared to high-temperature conventional thermal processing (105 °C for 10 min). Furthermore, beta-ecdysone stability and sugar hydrolysis also were evaluated.

## 2. Results and Discussion

### 2.1. Physicochemical Properties

Table 1 presents the pH values, and soluble solids content of the *Pfaffia glomerata* root aqueous extract-based functional beverage after non-thermal and thermal SC-CO_2_ treatments and conventional high-temperature processing in comparison to the untreated beverage. Both SC-CO_2_ treatments and thermal processing did not change the pH values and soluble solids content of the samples (*p*-value ≥ 0.089). Bertolini et al. [25] reported similar results for the pomegranate juice treated with SC-CO_2_ technology by employing process conditions from 8 to 16 MPa, 35 to 45 °C, and 20 to 40 min. They verified that the pH and soluble solids content of the juice did not show any changes after all treatments that were evaluated. Murtaza et al. [23] processed apple (*Malus domestica*) juice with CO_2_ at different phases (liquid, gas, critical, and supercritical) to inactivate polyphenol oxidase. The authors confirmed that the CO_2_ negligibly affected the pH and soluble solids content of the apple juice. Regarding conventional high-temperature thermal processing, the pH values and soluble solids content of concentrated apple juice processed in industrial-scale were stable after all thermal manufacturing stages such as preconcentration, enzymatic clarification, concentration, and pasteurization [26]. However, Amanina et al. [27] observed a slight increase in pH and soluble solids content of pineapple-mango juice blend after thermal processing using a batch pasteurizer at 90 °C for 5 min.

The maintenance of the pH values during the processing of food beverages is fundamental to ensure the chemical stability of bioactive compounds obtained from plant-materials. During SC-CO_2_ treatment, a small and temporary lowering of pH in the liquid system is observed due to the formation of carbonic acid; however, after the depressurization step, pH returns to its initial value [28]. Soluble solids content is an essential indicator of sugar content in fruit and vegetable juices. Significant changes in this physicochemical parameter may affect the organoleptic preference of juices. SC-CO_2_ technology has been recognized as an innovative stabilization technique mainly due to its performance to inactivate microorganisms and enzymes, nevertheless maintaining the physicochemical properties of plant-based beverages such as fruit and vegetable juices similar to untreated products [24].

### 2.2. Sugar Content

Sugar content has an essential role in the overall taste of fruit-based beverages because it contributes to the right balance between their sweetness and sourness provided by organic acids like citric and malic acid. Thus, sugar content influences the sensory attributes of these products. The evaluation of the monosaccharides and disaccharides content after high-energy treatments, such as thermal processing and high-pressure CO_2_, may assist in understanding how these treatments could be promoting undesired reactions of sugar degradation. A reduction of the disaccharides content followed by a monosaccharides content increase after those treatments could be associated with the hydrolysis of disaccharides and polysaccharides. Likewise, a reduction of both saccharides content could be associated with their degradation due to oxidation and Maillard reaction, resulting in changes in sensory, nutritional, and functional properties of food products rich or enriched with them [29].

The glucose, fructose, and sucrose content of the *Pfaffia glomerata* root aqueous extract-based functional beverage samples are shown in Table 2. The predominant sugars in the functional beverage are fructose, with approximately 64% of the total sugar content, followed by glucose (20%) and sucrose (16%), respectively. The SC-CO_2_ treatments did not modify the sugar content (*p*-value ≥ 0.08). However, conventional thermal processing promoted a reduction of 17% in sucrose content (*p*-value = 0.041). Regarding total sugar content, this reduction was less than 8% and did not affect the soluble solids content (Table 1). Wang et al. [30] evaluated quality changes of sugarcane juice after thermal treatments under different temperatures (90, 100, and 110 °C) and processing time (10, 20, and 30 s). The authors observed an increase of glucose content, whereas a sucrose content reduction was verified for all temperatures and processing time as well as their combinations. For fructose content, a maximum peak of concentration was found at 20 s for all temperatures. The sugar chemical stability to SC-CO_2_ processing has been reported in different fruit juices such as apple juice [31], strawberry juice [32], coconut water [33], and others. Furthermore, SC-CO_2_-treated juices have presented higher sugar chemical stability during the storage time in comparison to juices stabilized by other techniques. Pei et al. [34] studied the effects of SC-CO_2_ (30 MPa, 65 °C, and 15 min), high hydrostatic pressure (400 MPa, 45 °C, and 10 min) and thermal processing (85 °C and 10 min) on the chemical stability of sugars (glucose, fructose, and sucrose) in melon juice. Thermal processing promoted a significant reduction of all sugars evaluated in melon juice while SC-CO_2_ and high hydrostatic pressure preserved the sugar content. Also, SC-CO_2_-treated samples maintained their sucrose content during storage time (7, 14, 21, and 28 days), but the samples treated by the other stabilization techniques had their content decreased.

### 2.3. FOS Chemical Stability

The maintenance of the FOS integrity after stabilization treatments of food products enriched with them is an important issue for the development of novel prebiotic beverages. The functionality of fermentable dietary fiber to stimulate the growth/activity of beneficial bacteria aiming to modulate the gut microbiota and restore health in microbiota-linked diseases depends on their daily consumption. A supplementation with prebiotic carbohydrates equivalent to 9 g daily intake can induce a promising metabolic response [35]. Table 3 presents the FOS content regarding 1-kestose (GF_2_), nystose (GF_3_), and fructofuranosylnystose (GF_4_) of the functional beverage. GF_n_-type fructans are FOS with a linear structure of ‘n’ number of fructose units with a residual glucose molecule. The thermal SC-CO_2_ treatment had a significant effect on the increase of the GF_3_ (*p*-value = 0.014) and GF_4_ (*p*-value = 0.039) content, while the non-thermal treatment did not affect the FOS content. The pressure and CO_2_ volume ratio did not present a significant effect on these responses. The pressure range used in the SC-CO_2_ technique to stabilize food products is lower than that applied in high-pressure processing (HPP). SC-CO_2_ treatment works from 8 to 60 MPa, whereas HPP-stabilized products are subjected to pressure range from 100 to 1000 MPa. In the SC-CO_2_ technique, there is a synergic effect between CO_2_ and pressure to inactivate microorganisms and enzymes. Thus, a moderate pressure range is enough to promote stabilization effects when compared to HPP. However, the stabilization mechanisms associated with HPP are mainly based on mechanical energy [24]. Almeida et al. [12] reported a FOS hydrolysis of 21.4% in samples of FOS-enriched orange juice subjected to non-thermal HPP at 450 MPa for 5 min at 11.5 °C.

A reduction of 12% in GF_2_ content was observed (Table 3) after conventional thermal processing performed at 105 °C for 10 min (*p*-value = 0.021). FOS are liable to hydrolysis and depolymerization during thermal processing applied to fruit juices and beverages mainly due to low pH values of these liquid systems that, when assisted by high-temperatures, favors FOS degradation. Matusek et al. [16] evaluated the chemical stability of FOS in buffered solutions with pH between 2.7 and 3.3 subjected to thermal processing from 60 to 100 °C. They demonstrated that under acid conditions, the hydrolysis of FOS was insignificant at 60 °C; however, increasing the temperature from 70 to 80 °C, a considerable amount of them could be hydrolyzed. According to the authors, all of the oligomers were degraded after 1–1.5 h at 100 °C. Klewicki [36] studied the FOS stability during pasteurization of fruit juice and fruit-milk beverage at a low pH ranging from 2.7 to 4.2. The authors reported a FOS hydrolysis up to 80% and concluded that the amount of hydrolyzed FOS was higher at lower pH values when associated with longer pasteurization time. Chi et al. [37] studied the chemical stability of short-chain FOS obtained from *Morinda Officinalis*, with degrees of polymerization from three to nine, due to approval of their prescription for the treatment of mild and moderate depression episodes in China. They evaluated the GF_4_ stability at 37 °C in simulated gastric fluid (pH 1.2) using pepsin from porcine gastric mucosa. GF_4_ was hydrolyzed into GF_3_, GF_2_, sucrose, and fructose, suggesting that glycosides bonds are easily cleaved under strongly acidic conditions. After 60 min, the residual percentage of GF_4_ was only 11.9%.

**Figure 1:**
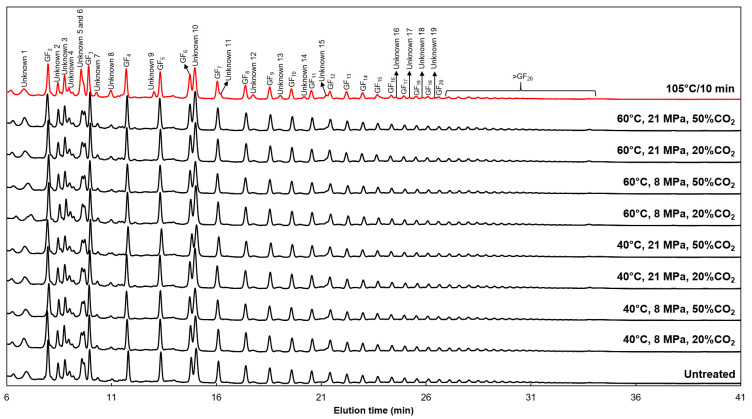
Effects of SC-CO_2_ treatments and conventional thermal processing on the chromatographic profile of the FOS-type fructans of the *Pfaffia glomerata* root aqueous extract-based functional beverage.

The increase in GF_3_ and GF_4_ contents, after thermal SC-CO_2_ treatment, can be associated with the hydrolysis of FOS with a degree of polymerization higher than that of fructans. The chromatographic profiles obtained by HPAEC-PAD in the *Pfaffia glomerata* root aqueous extract-based functional beverage before and after all treatments are shown in Figure 1. According to them, the eight most abundant peaks could be identified as GF_2_, GF_3_, GF_4_, GF_5_, GF_6_, GF_7_, GF_8_, and GF_9_. Also, other fructans from moderate to high degrees of polymerization were identified (GF_10_ to GF_20_). However, their peak areas reduced with the increase in their degree of polymerization. Table 4 presents a quantitative analysis from the analytical curves for all FOS molecules found in the functional beverage using their peak areas. Thus, the main FOS found in *Pfaffia glomerata* root aqueous extract were those with a degree of polymerization from 3 to 10. The low pH of the functional beverage (Table 1) associated with the temporary lowering of pH during SC-CO_2_ treatment at 60 °C favored the hydrolysis of FOS with moderate to a high degree of polymerization into GF_3_ and GF_4_ molecules. However, an accurate molecular balance between the hydrolysis of short-chain and long-chain FOS is very complicated because the *Pfaffia glomerata* root aqueous extract has FOS with different degrees of polymerization.

A combined analysis between Figure 1 and Table 4 evidenced the formation/rise of distinct peaks after conventional thermal processing, demonstrating that this high-energy treatment hydrolyzed the FOS molecular chain. These distinct peaks were identified as unknown compounds. Conventional thermal processing promoted the formation of four unknown compounds, such as unknown 11, 14, 15, and 19, and raised many unknown compounds, such as unknown 5, 7, 9, 12, 13, 16, 17, and 18 (Table 4). On the other hand, no distinct peak was formed after the non-thermal or thermal SC-CO_2_ treatments. Although the increase in GF_3_ and GF_4_ content attributed to thermal SC-CO_2_ treatment occurred by hydrolysis mechanisms, this innovative stabilization technique promoted the preservation of the chromatographic profile of FOS molecules. Similar results were observed by Silva et al. [38]. They studied the chemical stability of a commercial sample of inulin with a high degree of polymerization added in apple juice and subjected to thermal processing (95 °C/1 min) and non-thermal SC-CO_2_ treatment (35 °C, 10–20 MPa, and 10 min). The inulin molecular chain was broken into short-chain FOS units by the thermal treatment, whereas the SC-CO_2_ technique preserved inulin integrity besides antioxidant activity and other functional compounds found in apple juice. However, in their study, only a non-thermal approach for the SC-CO_2_ technology was evaluated.

### 2.4. Beta-Ecdysone Stability

Beta-ecdysone was highly stable for non-thermal and thermal SC-CO_2_ treatments and conventional thermal processing. Its content after all stabilization treatments applied to the *Pfaffia glomerata* roots aqueous extract-based functional beverage samples remained constant in comparison to the untreated beverage (*p*-value = 0.2). A content of 0.032 ± 0.001 mg/mL of beta-ecdysone was determined in all samples. Vardanega et al. [15] evaluated the chemical stability of beta-ecdysone extracted from *Pfaffia glomerata* roots after stabilization techniques to produce a functional powder tea from the aqueous extract. They studied the effects of freeze-drying at −40 °C for 60 h and spray-drying technique with the inlet and outlet temperatures of 180 and 120 °C, respectively. Beta-ecdysone was chemically stable after both drying techniques. They obtained the same content of beta-ecdysone in both reconstituted powder teas.

## 3. Materials and Methods

### 3.1. Obtaining Pfaffia Glomerata Root Aqueous Extract

*Pfaffia glomerata* roots powder was produced according to the methodology described by Vardanega et al. [39]. The plant material was collected in the experimental field of CPQBA (UNICAMP, Campinas, Brazil). Then, the roots were washed and dried at 40 °C for 5 days in a forced air circulation dryer. Dried roots were comminuted in a knife mill (Tecnal, model TE 631, Piracicaba, Brazil), and a mean diameter of 8 µm was obtained. *Pfaffia glomerata* root aqueous extract was produced by using low-pressure water extraction at 60 °C and ambient atmospheric pressure. The experimental unit used for the extractions was described by Vardanega et al. [40]. A 450-mL reactor was loaded with 150 g of powdered roots. The water mass to *Pfaffia glomerata* powder mass ratio used was 3.7, and the water flow rate was 10 mL/min. The aqueous extract presented a soluble solids content of 2.5 ± 0.1%.

### 3.2. Functional Beverage Formulation

The *Pfaffia glomerata* aqueous extract-based functional beverage was formulated using apple juice as a carrier system of the FOS and beta-ecdysone. Clarified apple juice concentrate with approximately 70% soluble solid content was purchased from Fischer S/A Agroindústria (Videira, Brazil). The beverage was produced according to the following mass balance (g/100 g beverage): 20 g *Pfaffia glomerata* aqueous extract, 20 g clarified apple juice concentrate; and 60 g distilled water (Millipore^®^). The functional beverage presented a soluble solids content of 14.9 ± 0.1%. Beverage samples used as control were named “untreated.”

### 3.3. Supercritical CO_2_ Processing

The functional beverage was processed using the SC-CO_2_ unit described and validated by Silva et al. [41]. According to them, this unit was able to inactivate up to 6.6 log cycles of *Lactobacillus casei* cells. They studied a range of pressure from 10 to 20 MPa, temperature from 35 to 55 °C, processing time from 10 to 30 min, and the CO_2_ volume ratio from 10 to 70%. The percentage of CO_2_ volume was evaluated concerning the total volume of the high-pressure reactor. The authors also described the steps for the processing. A 630 mL high-pressure cylinder reactor with a height of 240 mm and an inner diameter of 67.85 mm was used. CO_2,_ with a purity ≥ 99.9%, was purchased from Gama Gases Especiais LTDA (São Bernardo do Campo, Brazil). A thermostatic bath (Marconi, MA-184, Piracicaba, Brazil) was used to cool the CO_2_ to −4 °C and a pneumatic pump (Maximator, M-111 L, Nordhausen, Germany) for the CO_2_ pressurization.

The effects of temperature (40 and 60 °C), pressure (8 and 21 MPa), and CO_2_ volume ratio (20 and 50%) on the pH, soluble solids content, glucose, fructose, sucrose, 1-kestose, nystose, and fructofuranosylnystose, and beta-ecdysone content were evaluated using a full factorial experimental design (2 × 2 × 2). These experiments were carried out with a processing time of 10 min after the system reached the working pressure. All experiments were performed in duplicate.

### 3.4. Conventional Thermal Treatment

The *Pfaffia glomerata* aqueous extract-based functional beverage also was thermally treated at 105 °C for 10 min in an autoclave (Fabbe Primar Industrial LTDA, Model 146, São Paulo, Brazil). Three hundred milliliters of beverage were treated inside a 500 mL glass bottle. The initial temperature of the samples was 30 °C. The thermal treatment was carried out in duplicate.

### 3.5. pH and Soluble Solids Content Analysis

The pH analysis was carried out in duplicate at 25 ± 1 °C using a DIGMED DM-22, V 1.2 pH meter (Digicrom Analytical, São Paulo, Brazil). The soluble solids content was measured with a digital refractometer model PAL-1 (Atago Brasil LTDA, Ribeirão Preto, Brazil).

### 3.6. HPAEC-PAD Sugars and FOS Analysis

The analysis of sugars and FOS in the beverages was performed by High-Performance Anion Exchange Chromatography coupled with Pulsed Amperometric Detection (HPAEC-PAD) using an ion chromatographer Dionex ICS-5000 (Thermo Fisher Scientific, Waltham, MA, USA) according to the method described by Silva et al. [38]. Sugars (glucose, fructose, and sucrose) were separated by isocratic elution using 0.12 M NaOH on a Carbopac PA1 column (250 × 4 mm i.d., particle size 10 μm, Thermo Fisher Scientific, Waltham, MA, USA). In contrast, FOS was separated by gradient elution using three mobile phases: solution A (0.2 M NaOH), solution B (1 M sodium acetate containing 0.2 M NaOH) and solution C (ultrapure water) on a Carbopac PA100 column (250 × 4 mm i.d., particle size 8.5 μm, Thermo Fisher Scientific, Waltham, MA, USA). The gradient was performed as follows: 0–2 min, 48.5% A and 1.5% B; 2–44 min, 48.5–0% A and 1.5–50% B; 44–49 min, 50% B; and 49–54 min, 48.5% A and 1.5% B (the solution C was maintained at 50% along the chromatographic run). In both analyses, the samples were diluted in ultrapure water, filtered through a 0.22 μm PTFE filter and injected into the column using an auto-sampler. The column temperature was maintained at 30 °C, the flow rate was 1.0 mL/min, and the injection volume of the samples was 25 μL. Data were acquired and processed using Chromeleon software version 7.0. The sugars (glucose, fructose, and sucrose) and FOS (1-kestose, nystose, and fructofuranosylnystose) were identified in samples by comparing the retention times of authentic standards and the samples. Calibration curves were constructed with commercial standards (0.25–12.50 µg/mL) to quantify the sugars/FOS in the juices. The content of individual sugars and FOS was expressed as mg/mL and µg/mL juice, respectively. From GF_5_ to GF_20_, the identification of the fructans was performed by analyzing their eluotropic order in comparison to an inulin standard, as described by Arruda et al. [29].

### 3.7. HPLC-DAD Beta-Ecdysone Analysis

The analysis of beta-ecdysone content in the functional beverage samples was carried out by high-performance liquid chromatography coupled to a diode array detector (HPLC-DAD) using a Waters (Alliance, Milford, MA, USA) chromatographer. The methodology described by Rostagno et al. [42] for the identification and quantification of beta-ecdysone was reproduced in this study. Data acquisition was performed at 246 nm using as standard 20-hydroxyecdysone (beta-ecdysone) purchased from Sigma Aldrich (St. Louis, MO, USA).

### 3.8. Statistical Analysis

The effects of the SC-CO_2_ process conditions (temperature, pressure and CO_2_ volume ratio) and conventional thermal treatment on the sugars, FOS, and beta-ecdysone content were evaluated by analysis of variance (ANOVA) using Minitab 18^®^ software at a significance level of 5% (*p*-value < 0.05).

## 4. Conclusions

SC-CO_2_ technology is a promising alternative for the processing of prebiotic carbohydrate-enriched beverages like FOS-type fructans. The supplementation of apple juice with *Pfaffia glomerata* roots aqueous extract provided an innovative functional beverage due to its high content of short-chain FOS and beta-ecdysone. FOS-type fructans with the degree of polymerization from 3 to 10 were chemically stable to SC-CO_2_ processing from non-thermal (40 °C) to thermal (60 °C) treatment. Different process conditions of pressure and CO_2_ volume ratio did not influence the FOS content and their chemical profile, however, increasing the temperature from 40 to 60 °C promoted an increase in the short-chain FOS content with the degree of polymerization four and five (nystose and fructofuranosylnystose, respectively). High-temperature thermal processing (105 °C for 10 min) favored the hydrolysis of the FOS with the degree of polymerization three (1-kestose) and reduced the sucrose content. Also, this high-energy treatment degraded the FOS molecular structure, increasing the content of unknown compounds and promoting the formation of other ones, whereas SC-CO_2_ treatments did not alter the chemical profile of FOS added to the beverage. Regarding beta-ecdysone, its content remained constant after all stabilization treatments demonstrating thus its high chemical stability.

## Figures and Tables

**Table 1:** Effects of supercritical carbon dioxide (SC-CO_2_) treatments and conventional thermal processing on the physicochemical properties of the *Pfaffia glomerata* root aqueous extract-based functional beverage (*n* = 6).

Treatment	Pressure (MPa)	CO_2_ Volume Ratio (%)	pH (-)	Soluble Solids Content (%)
**Non-thermal**	8	20	4.12 ± 0.02	14.9 ± 0.1
**(40 °C)**		50	4.10 ± 0.01	14.7 ± 0.1
	21	20	4.12 ± 0.01	14.9 ± 0.1
		50	4.12 ± 0.01	14.7 ± 0.1
**Thermal**	8	20	4.02 ± 0.01	14.6 ± 0.1
**(60 °C)**		50	4.02 ± 0.01	14.9 ± 0.1
	21	20	4.0 ± 0.1	14.9 ± 0.1
		50	4.0 ± 0.1	14.9 ± 0.1
**105 °C/10 min**	-	-	4.1 ± 0.1	14.6 ± 0.3
**Untreated**	-	-	4.1 ± 0.1	14.8 ± 0.1

**Table 2:** Effects of SC-CO_2_ treatments and conventional thermal processing on the sugar content of the *Pfaffia glomerata* root aqueous extract-based functional beverage (*n* = 6).

Treatment	Pressure (MPa)	CO_2_ Volume Ratio (%)	Glucose (mg/mL)	Fructose (mg/mL)	Sucrose (mg/mL)	Total Sugars (mg/mL)
**Non-thermal**	8	20	29 ± 1	96 ± 2	24 ± 1	149 ± 1
**(40 °C)**		50	28 ± 2	91 ± 11	23 ± 2	141 ± 14
	21	20	27 ± 3	89 ± 9	22 ± 3	139 ± 15
		50	27 ± 1	88 ± 5	21 ±1	135 ± 7
**Thermal**	8	20	28 ± 1	92 ± 2	23 ±1	143 ± 4
**(60 °C)**		50	30 ± 1	99 ± 3	24 ± 1	153 ± 3
	21	20	29 ± 1	93 ± 1	24 ± 1	146 ± 1
		50	30 ± 2	96 ± 7	24 ± 1	150 ± 11
**105 °C/10 min**	-	-	27 ± 2	88 ± 6	19 ± 1	134 ± 9
**Untreated**	-	-	29 ± 1	94 ± 1	23 ± 1	146 ± 1

**Table 3:** Effects of SC-CO_2_ treatments and conventional thermal processing on the fructooligosaccharide (FOS) content of the *Pfaffia glomerata* root aqueous extract-based functional beverage (*n* = 6).

Treatment	Pressure (MPa)	CO_2_ Volume Ratio (%)	GF_2_ (µg/mL)	GF_3_ (µg/mL)	GF_4_ (µg/mL)	Total FOS (µg/mL)
**Non-thermal**	8	20	324 ± 11	236 ± 4	269 ± 3	828 ± 4
**(40 °C)**		50	309 ± 21	221 ± 8	252 ± 15	782 ± 44
	21	20	322 ± 5	237 ± 9	255 ± 13	815 ± 9
		50	308 ± 22	225 ± 15	255 ± 21	788 ± 58
**Thermal**	8	20	296 ± 11	236 ± 5	264 ± 4	797 ± 20
**(60 °C)**		50	313 ± 8	241 ± 2	272 ± 4	827 ± 13
	21	20	292 ± 27	242 ± 7	270 ± 2	804 ± 19
		50	294 ± 23	248 ± 3	274 ± 2	816 ± 22
**105 °C/10 min**	-	-	268 ± 1	234 ± 13	251 ± 14	753 ± 28
**Untreated**	-	-	306 ± 8	228 ± 6	259 ± 7	794 ± 5

**Table 4:** Effects of SC-CO_2_ treatments and conventional thermal processing on the FOS molecular profile regarding their peak area (*n*C × min) measured at the same dilution of a hundred times.

Compound	Retention Time (min)	Untreated	Thermal Processing (105 °C/10 min)	SC-CO_2_ Treatment							
				Non-Thermal (40 °C)				Thermal (60 °C)			
				8 MPa 20%CO_2_	8 MPa 50%CO_2_	21 MPa 20%CO_2_	21 MPa 50%CO_2_	8 MPa 20%CO_2_	8 MPa 50%CO_2_	21 MPa 20%CO_2_	21 MPa 50%CO_2_
Unknown 1	6.91	3.33	2.86	3.49	3.05	2.98	3.06	3.14	3.03	3.08	3.02
GF_2_	7.97	7.43	6.17	7.52	6.66	7.36	6.65	7.17	6.86	7.02	6.69
Unknown 2	8.46	2.82	2.08	2.87	2.61	2.87	2.61	2.85	2.84	2.78	2.88
Unknown 3	8.78	2.64	2.61	2.82	2.44	2.33	2.50	2.64	2.71	2.58	2.59
Unknown 4	8.99	0.71	0.48	0.63	0.64	0.67	0.63	0.73	0.82	0.75	0.73
Unknown 5	9.59	3.92	5.30	2.55	2.45	2.36	2.61	2.97	3.24	3.58	3.13
Unknown 6	9.71	2.54	1.55	2.63	2.18	2.33	2.09	2.29	2.10	2.30	2.48
GF_3_	9.96	5.06	4.97	5.34	4.73	5.59	4.71	5.39	5.42	5.45	5.39
Unknown 7	10.33	0.58	0.83	0.59	0.36	0.40	0.56	0.63	0.65	0.63	0.75
Unknown 8	10.99	0.54	1.76	0.65	0.74	0.71	0.74	1.02	1.03	1.02	1.04
GF_4_	11.75	5.02	5.21	5.36	4.76	4.89	4.75	5.35	5.45	5.41	5.44
Unknown 9	13.04	0.03	0.94	0.04	0.03	0.04	0.03	0.06	0.08	0.07	0.07
GF_5_	13.33	5.37	4.69	5.63	5.02	4.99	5.00	5.65	5.73	5.72	5.73
GF_6_	14.76	4.75	5.17	5.11	4.30	4.62	4.45	5.14	5.03	5.29	5.61
Unknown 10	15.00	8.34	7.30	8.84	7.71	7.72	7.97	9.60	9.71	9.83	10.13
GF_7_	16.07	4.36	3.56	4.58	4.06	4.02	4.02	4.49	4.54	4.52	4.53
Unknown 11	16.25	n.d.	0.70	n.d.	n.d.	n.d.	n.d.	n.d.	n.d.	n.d.	n.d.
GF_8_	17.40	3.77	2.96	3.92	3.51	3.49	3.49	3.91	3.98	3.97	3.98
Unknown 12	17.76	0.39	1.09	0.36	0.33	0.33	0.32	0.37	0.41	0.39	0.41
GF_9_	18.56	3.34	2.60	3.51	3.11	3.08	3.10	3.47	3.52	3.52	3.52
Unknown 13	18.98	0.40	1.04	0.39	0.35	0.36	0.33	0.42	0.46	0.45	0.44
GF_10_	19.60	2.99	2.20	3.13	2.77	2.74	2.77	3.09	3.14	3.11	3.13
Unknown 14	20.18	n.d.	0.67	n.d.	n.d.	n.d.	n.d.	n.d.	n.d.	n.d.	n.d.
GF_11_	20.56	2.61	1.82	2.78	2.47	2.41	2.46	2.77	2.79	2.76	2.79
Unknown 15	21.25	n.d.	0.50	n.d.	n.d.	n.d.	n.d.	n.d.	n.d.	n.d.	n.d.
GF_12_	21.43	2.52	1.51	2.77	2.46	2.49	2.47	2.76	2.83	2.83	2.81
GF_13_	22.24	1.90	1.64	1.99	1.77	1.74	1.76	1.96	2.00	1.98	1.98
GF_14_	22.98	1.63	1.34	1.71	1.51	1.49	1.51	1.69	1.72	1.71	1.71
GF_15_	23.69	1.28	1.10	1.32	1.17	1.15	1.18	1.32	1.32	1.31	1.33
GF_16_	24.35	1.08	0.50	1.15	0.99	1.01	1.01	1.14	1.13	1.13	1.11
Unknown 16	24.61	0.05	0.14	0.05	0.05	0.05	0.05	0.06	0.06	0.06	0.06
GF_17_	24.97	0.96	0.54	1.02	0.89	0.89	0.89	0.99	1.00	1.01	1.01
Unknown 17	25.24	0.05	0.18	0.06	0.05	0.05	0.05	0.06	0.06	0.06	0.06
GF_18_	25.55	0.84	0.46	0.89	0.79	0.78	0.80	0.89	0.89	0.89	0.90
Unknown 18	25.83	0.04	0.14	0.04	0.03	0.03	0.04	0.04	0.04	0.04	0.04
GF_19_	26.10	0.73	0.35	0.76	0.67	0.66	0.67	0.75	0.74	0.74	0.75
Unknown 19	26.43	n.d.	0.12	n.d.	n.d.	n.d.	n.d.	n.d.	n.d.	n.d.	n.d.
GF_20_	26.61	0.63	0.35	0.66	0.57	0.57	0.58	0.66	0.73	0.65	0.74

n.d.: not detected.
